# Fabrication of Pt/Ti/TiO_2_ Photoelectrodes by RF-Magnetron Sputtering for Separate Hydrogen and Oxygen Production

**DOI:** 10.3390/ma9040279

**Published:** 2016-04-08

**Authors:** Gian Luca Chiarello, Cristina Tealdi, Piercarlo Mustarelli, Elena Selli

**Affiliations:** 1 Dipartimento di Chimica, Università degli Studi di Milano, via Golgi 19, Milano 20133, Italy; elena.selli@unimi.it; 2 Dipartimento di Chimica, Università degli Studi di Pavia, viale Taramelli 16, Pavia 27100, Italy; cristina.tealdi@unipv.it (C.T.); piercarlo.mustarelli@unipv.it (P.M.)

**Keywords:** photocatalytic hydrogen production, water splitting, TiO_2_ thin film, RF-magnetron sputtering

## Abstract

Evolution of pure hydrogen and oxygen by photocatalytic water splitting was attained from the opposite sides of a composite Pt/Ti/TiO_2_ photoelectrode. The TiO_2_ films were prepared by radio frequency (RF)-Magnetron Sputtering at different deposition time ranging from 1 up to 8 h and then characterized by X-ray diffraction (XRD), scanning electron microscopy (SEM) and ultraviolet-visible-near infrared (UV-vis-NIR) diffuse reflectance spectroscopy. The photocatalytic activity was evaluated by incident photon to current efficiency (IPCE) measurements and by photocatalytic water splitting measurements in a two-compartment cell. The highest H_2_ production rate was attained with the photoelectrode prepared by 6 h-long TiO_2_ deposition thanks to its high content in the rutile polymorph, which is active under visible light. By contrast, the photoactivity dropped for longer deposition time, because of the increased probability of electron-hole recombination due to the longer electron transfer path.

## 1. Introduction

Photocatalytic water splitting into hydrogen and oxygen is one of the most promising ways to produce a renewable and environmentally friendly fuel by converting solar into chemical energy. A separate stream of pure hydrogen can be attained using two-compartment photocatalytic cells [1,2,3,4,5,6,7]. The main issue in the development of such devices is the preparation of photoactive, stable, and cost effective photoanodes in the form of thin layers. Among all of the existing techniques for thin films deposition [8], radio frequency (RF) magnetron sputtering proved to be an effective method to prepare photoactive semiconductor coatings [4,9,10,11]. Moreover, this technique is already available for large surface deposition and scale production (e.g., roll-to-roll magnetron sputtering). A precise control of all the deposition parameters (RF power, total pressure inside the chamber, deposition time, temperature and distance of the substrate) allows one to tune the characteristics of the deposited film. For example, Ebrahimi *et al.* [12] showed that the crystal phase composition of a TiO_2_ film can be changed from pure rutile to a mixture of anatase and rutile by increasing the total Ar pressure during deposition. In a previous work [4], we showed that almost pure anatase is obtained by keeping the substrate temperature at 450 °C, whereas prevalently rutile is obtained at 600 °C. Furthermore, both cation (e.g., Cr, Fe, V and Cu) [13,14,15] or anion (N and/or C) [16,17,18] doping of the TiO_2_ coatings have been reported by co-deposition or reactive magnetron sputtering, respectively. Chemical post treatment with HF etching [19] or hydrothermal treatment with NaOH [20] have also been reported to increase the photocatalytic performance.

In this work, we present the photocatalytic water splitting results obtained employing composite Pt/Ti/TiO_2_ photoelectrodes prepared by RF magnetron sputtering, which allow the cleavage of H_2_O into H_2_ and O_2_ on the two opposite sides upon illumination. In particular, we focus on the effects that the deposition time has on the properties of the TiO_2_ coating and on its photocatalytic performance.

## 2. Materials and Methods

### 2.1. Preparation of the Photoelectrodes

TiO_2_ thin films (9.6 cm^2^) were deposited on pure titanium disks (TI000420/8, Goodfellow, Huntingdon, UK) starting from a TiO_2_ powder target (Puratronic, 99.995%, Alfa Aesar, Haverhill, MA, USA), employing a RF magnetron sputtering system (Rial Vacuum, Chiozzola, Italy). Prior to deposition, titanium disks were immersed for 1 min in a pickling aqueous solution (DeTitan 90, Kemar, Leggiuno, Italy) containing HF, H_2_SO_4_ and H_2_O_2_. All depositions were carried out in pure Ar at constant sputtering power (200 W) and total gas pressure inside the chamber (2.0 Pa). The Ti disk substrate was placed at a distance of 50 mm above the TiO_2_ target and kept at 600 °C during the deposition. Four samples were prepared at different deposition times (1, 3, 6 and 8 h).

A thin platinum coating (*ca.* 20 mg·cm^−2^ loading) was deposited on the opposite side of all titanium disks in a vacuum evaporator, finally obtaining the Pt/Ti/TiO_2_ photoelectrodes.

### 2.2. Characterization of the Photoelectrodes

The film morphology was investigated by scanning electron microscopy (SEM) employing a LEO 1430 microscope (Zeiss, Jena, Germany). X-ray diffraction (XRD) patterns of the deposited TiO_2_ coatings were acquired with a PW3020 powder diffractometer (Philips, Amsterdam, The Netherlands), using the Cu Kα radiation (λ = 1.5418 Å) in the 20°–80° 2θ range with a time step of 0.05° and a fixed counting time of 2 s per step. Quantitative phase analysis was made by the Rietveld refinement method [21,22], using the “Quanto” software [23]. UV-Vis-NIR diffuse reflectance (DR) spectra were recorded in the 220 nm < λ < 2600 nm range with a UV3600 Plus spectrophotometer (Shimadzu, Kyoto, Japan) equipped with an ISR-603 integrating sphere.

### 2.3. IPCE Measurements

Incident photon to current efficiency (IPCE) curves were measured with a homemade single compartment Plexiglas cell with a Pyrex glass window by connecting the irradiated TiO_2_ film (anode) with a platinum counter electrode (cathode) through an external circuit including a DMM4040 digital multimeter (Tektronix, Beaverton, OR, USA) for photocurrent measurement. For these tests the Pt coating was covered by pressing the Ti disk against a silicone rubber foil. A 300 W Xe lamp with a Omni-λ 150 monochromator (LOT-Oriel, Darmstadt, Germany) was used as monochromatic irradiation source. The photocurrent was measured in a 1.0 M NaOH electrolyte solution without any external applied voltage in the 300–500 nm wavelength range with a 2 nm step and a 4 s time per step. The incident light power was measured with the same scan parameters using a S130VC calibrated photodiode (Thorlabs, Newton, NJ, USA) with the Pyrex window placed between the light source and the photodiode, to account for the transmittance of the cell window. The percent IPCE at each wavelength was calculated with the following formula:

(1) $$\%IPCE=\;\frac{I_{\lambda }\;\times 1240}{P_{\lambda }\times \lambda }\times 100$$

where *I*_λ_ is the photocurrent density (mA·cm^−2^); *P*_λ_ is the incident power density (mW·cm^−2^); λ (nm) is the incident wavelength; and 1240 (J·nm·C^−1^) = *h·c·e*^−1^ (*h* being the Planck constant, *c* the speed of light and *e* the charge of a single electron).

### 2.4. Separate H_2_ and O_2_ Photocatalytic Production Tests

**Figure 1:**
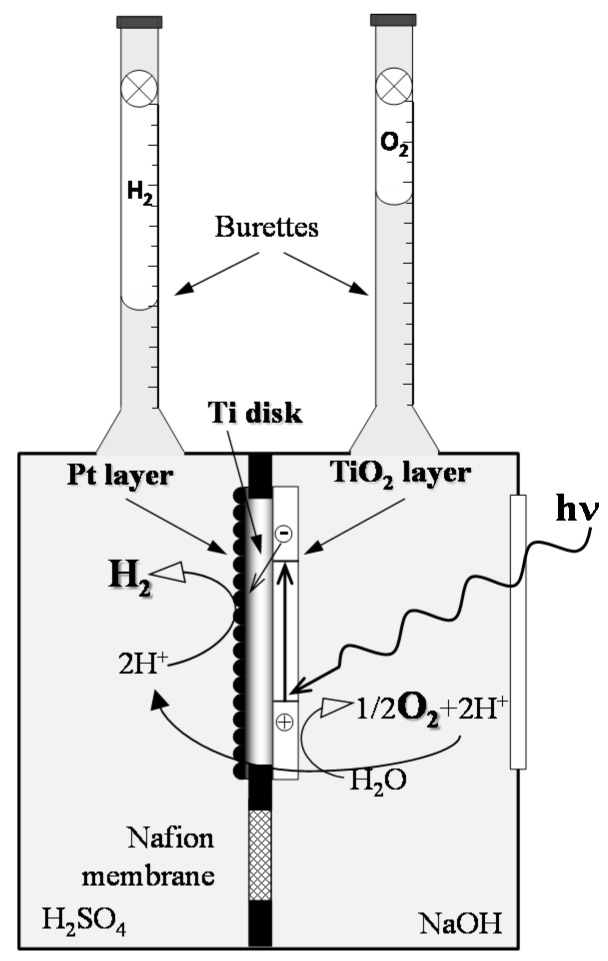
Schematic representation of the two-compartment photocatalytic cell.

The thus-obtained photoelectrodes were tested in a two-compartment photocatalytic Plexiglas cell, including a Pyrex glass optical window, which allows the separate evolution of pure hydrogen and oxygen from the aqueous solutions contained in the two compartments (Figure 1).

The cell has been fully described elsewhere [4]. The photoactive electrode was placed within the frame separating the two compartments, which were filled with 1.0 M NaOH and 0.5 M H_2_SO_4_ aqueous solutions, so that the illuminated anodic oxide coating was in contact with the alkaline solution, whereas the cathodic Pt-coated side of the titanium disk faced the acidic solution. Thus, a chemical bias was produced to assist the transfer of photopromoted electrons from the TiO_2_ film toward the Pt-coated side of the photoelectrode (Figure 1). The two solutions were separated by a Nafion 117 cation exchange membrane placed below the photoelectrode.

During irradiation, the evolved gases were collected into the two upside-down graduated burettes surmounting each cell compartment. The amounts of H_2_ and O_2_ were determined from the displacement of the solutions within each burette. The composition of the evolved gases was determined by gas-chromatographic analysis, after having sampled them with a gas-tight syringe. The irradiation source, which was switched on 15 min prior to the beginning of the runs, was a UV-vis iron halogenide mercury arc lamp (HG200, 250 W, Jelosil, Vimodrone, Italy) emitting in the 350 nm < λ < 450 nm range, with a full irradiation power density on the sample of *ca.* 19.7 mW·cm^−2^. The emission spectrum of the lamp was measured with a compact CCD (charge-coupled device) spectrometer (CCS100, Thorlabs, Newton, NJ, USA).

## 3. Results and Discussion

### 3.1. XRD and SEM Investigation

**Figure 2:**
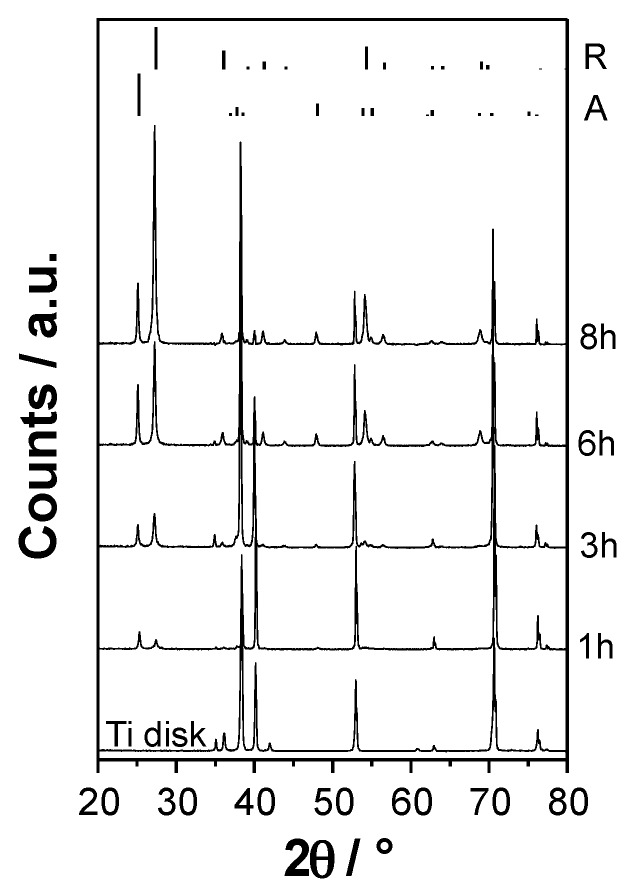
XRD patterns of the pristine Ti disk and of the TiO_2_ coatings grown for different deposition time (1, 3, 6, and 8 h). The peak positions and relative intensities of the anatase (A) and rutile (R) phases are reported at the top of the figure for comparison.

Figure 2 shows the XRD patterns of the investigated TiO_2_ films deposited in pure Ar at the same substrate temperature (600 °C) for different deposition times (1, 3, 6 and 8 h) together with that of the pristine metal Ti disk.

All RF magnetron sputtered coatings were composed of a mixture of anatase and rutile. The intensity of the main reflections of the two phases (2θ *ca.* 25° and *ca.* 27.5° for anatase and rutile, respectively) increased with an increase in the deposition time, while those of the metal Ti support decreased because of the growth of the TiO_2_ film thickness. The sample obtained after 1 h deposition was composed of *ca.* 54% anatase and 46% rutile, whereas the amount of rutile increased to *ca.* 70% for longer deposition time (Table 1). No evidence of the brookite polymorph was ever detected. A similar phase composition has been previously reported for coatings prepared by RF magnetron sputtering at a substrate temperature of 600 °C and Ar pressure of 2.0 Pa [12]. Moreover, the relative intensity of the reflection of the rutile phase at 2θ = 27.5° (corresponding to the (110) reflection) with respect to those at 2θ = 36.1° (101) and 2θ = 54.3° (211) is much larger compared to the reference one reported on top of Figure 2. This suggests that, during the sputtering process, the particles grow along a preferred orientation on the Ti support.

**Figure 3:**
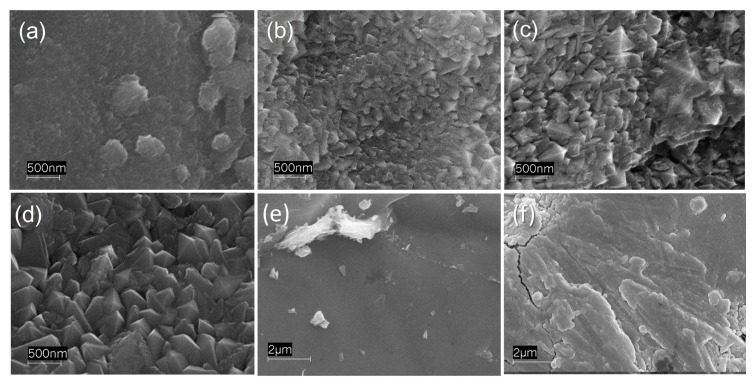
SEM images of TiO_2_ coatings prepared by radio frequency (RF) magnetron sputtering with (**a**) 1 h; (**b**) 3 h; (**c**) 6 h; and (**d**) 8 h deposition times; (**e**) Surface of the bare metal Ti disk surface; and (**f**) Pt film deposited on the back side of the titanium disk.

The surface morphology of the films was analyzed by SEM. The sample prepared after 1 h-long deposition (Figure 3a) shows a relatively smooth surface with some irregularities mostly arising from the Ti substrate underneath (Figure 3e).

By contrast, well defined TiO_2_ particles with pyramidal shape form after longer deposition time. During the evaporation process, the sputtered atoms, possessing relatively high kinetic energy, gradually add to the crystals growing on the substrate. According to the Wulff construction principle [24], the crystals assume the most thermodynamically stable shape in order to minimize the surface energy. Both anatase [24,25] and rutile [26] single crystals show a pyramidal shape.

Finally, the SEM images show that the particle size, and consequently the surface roughness, considerably increases from some tens to some hundreds of nanometers with an increase of the deposition time. Thus, the deposition time affects not only the thickness of the TiO_2_ film, but also its surface morphology, particle size and crystallinity.

### 3.2. UV-vis-NIR Diffuse Reflectance and IPCE Measurements

**Figure 4:**
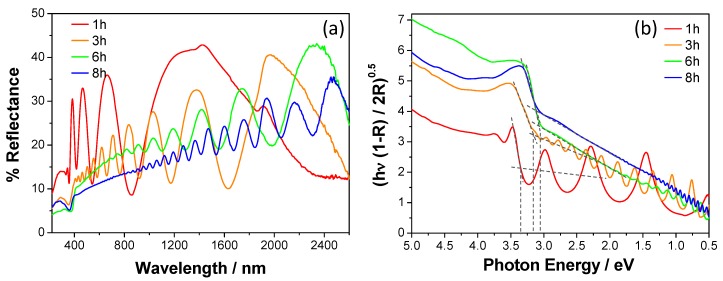
UV-vis-NIR diffuse reflectance spectra of the TiO_2_ coatings on a metal Ti disk for different deposition time. (**a**) Percent reflectance *vs.* wavelength spectra; and (**b**) corresponding Kubelka-Munk transform used for band gap calculation.

The UV-vis-NIR DRS spectra of all investigated samples exhibit the interference fringes typical of thin films in the vis-NIR region (Figure 4). These fringes originate from the interference of the waves reflected by the upper surface with those penetrating the film and reflected by the bottom surface. The frequency and the amplitude of the fringes depend on the film thickness. In particular, Figure 4 shows that the frequency increases and the amplitude decreases with an increase in the deposition time of the coatings (*i.e.*, an increase in the film thickness). The fringes are expected to vanish for very thick films, depending on the penetration depth of the incident light through the film itself.

The interference fringes are relevant in the characterization of thin films because they allow the evaluation of the film thickness [5]. In this work, we have used a dedicated tool of the spectrophotometer software and the obtained values are collected in Table 1. As expected, the thickness linearly increases as a function of the deposition time with a growth rate of *ca.* 350 nm·h^−1^.

The semiconductor absorption edges of the films are located below 420 nm. The band gap can be calculated from the Kubelka-Munk transform spectra (Figure 4b). This is usually done by taking the value at the intersection between a straight line interpolating the absorption edge and the abscissa axis, reporting the photon energy. However, the investigated films possess a very low reflectance in the vis-NIR region that linearly decreases towards lower wavelengths, likely due to the surface roughness of the films and the presence of the interference fringes. Consequently, all spectra appear shifted at higher [f(R)hν]^0.5^ values. Thus, band gap values have been here evaluated as the photon energy value at the crossing point between the straight line interpolating the portion of the spectrum at energy lower than the absorption edge (*i.e.*, the background) and the straight line interpolating the absorption edge (see dotted lines in Figure 4b). The band gap of bulk anatase and rutile notoriously are 3.2 eV and 3.0 eV, respectively. However, slightly larger values have been obtained for the coatings prepared after 1 h-long (3.30 eV) and 3 h-long (3.18 eV) deposition, despite of their high rutile content, whereas the same band gap of 3.05 eV has been obtained for those prepared by 6 h- and 8 h-long deposition, in line with the band gap of bulk rutile. It is known that the band gap of a semiconductor material can be affected by the particle size due to the confinement of the movement of electrons. The XRD and SEM investigations revealed that the deposition time affects not only the film thickness but also the crystal size. Thus, the unexpected larger band gap values of the two thinner TiO_2_ coatings (*i.e.*, 1 h- and 3 h-long deposited films) can be attributed to the size quantization effects that can widen the semiconductor band gap (*i.e.*, blue shift the absorption threshold) of small nanoparticles [27,28,29].

**Figure 5:**
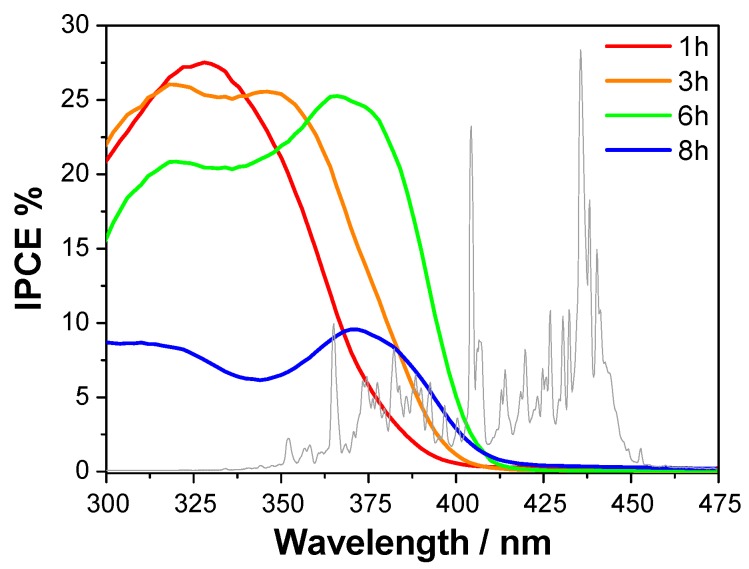
Effect of the deposition time on the incident photon to current efficiency (IPCE) curves of the TiO_2_-based photoelectrodes as a function of the incident wavelength. The emission spectrum of the Hg vapor lamp used as light source in separate H_2_ and O_2_ evolution photocatalytic tests is also shown (gray line).

The IPCE curves (Figure 5) show that the photoactivity threshold of the investigated series of samples progressively red-shifts with an increase in the coating thickness, in agreement with the calculated band gaps. In particular, the films prepared after 6 h- and 8 h-long deposition exhibit photoactivity above 390 nm, *i.e.*, in the visible region.

Two efficiency maxima can be distinguished in the IPCE curves, whereas curves measured on films of pure anatase or pure rutile usually show a single maximum (e.g., [5]). Thus, the first IPCE maximum, located around 325 nm, can be attributed to the anatase phase, while the second one, located at longer wavelengths (it appears as a shoulder in the 1 h-long deposited sample) can be attributed to the rutile phase. The latter maximum red-shifts together with the photoactivity edge.

It is interesting to note that the maximum IPCE is only partially related to the film thickness. Indeed, the first three photoelectrodes (deposition time ≤ 6 h) reach similar maximum efficiency values up to 27.5%, whereas IPCE considerably drops below 10% for the thickest one. In fact, increasing the semiconductor film thickness might lead to an increase in the fraction of the absorbed incident photons up to a boundary limit. On the other hand, the thicker the film is, the longer the electron transfer path is, with the consequent increase of electron-hole recombination probability. This latter phenomenon explains the sudden efficiency drop of the 8 h-long deposited sample.

### 3.3. Photocatalytic Water Splitting Test

The photocatalytic activity under polychromatic irradiation is strictly related to the IPCE curve, the emission spectrum of the light source, the incident power, and, in this case, the chemical bias (*i.e.*, the ΔpH between the two compartments of the photocatalytic cell).

**Figure 6:**
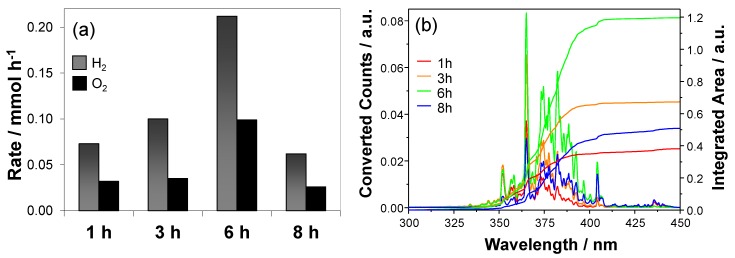
(**a**) Rate of hydrogen and oxygen production under polychromatic light irradiation obtained with the photoelectrodes obtained with different deposition times; (**b**) expected portion of converted incident spectrum and corresponding integrated area (calculated as the product of the IPCE curves times the emission spectrum of the light source).

Hydrogen and oxygen evolution occurred at a constant rate during irradiation, according to a pseudo zero order kinetics. The production rates obtained with the investigated series of photoelectrodes are listed in Table 2 and shown in Figure 6a.

As expected by the IPCE curves, the photocatalytic performance increased with an increase of the film thickness, reaching a maximum with the sample prepared after 6-h long deposition (0.21 mmol_H_2__·h^−1^, corresponding to *ca.* 5.3 NL h^−1^ per square meter of irradiated photoelectrode area) and then dropped for the thickest film. The increase of hydrogen production rate, *r*_H_2__, with increasing film thickness is clearly paralleled by a progressive red shift of the IPCE curves that allows the absorption and conversion of a larger portion of the incident light spectrum, as shown in Figure 6b.

This result is in line with the larger amount of the rutile phase having a narrower band gap with respect to anatase. The evolved hydrogen to oxygen ratio is slightly higher than the stoichiometric value of 2, as already observed and discussed recently [5].

Finally, two different types of photocatalytic efficiency in hydrogen production can be calculated. The first one is the expected efficiency, φ_exp_, calculated from the IPCE curves and the light emission spectrum, as:

φ_exp_ = *A*_conv._/*A*_i.s._ × 100 (2)

where *A*_conv._ and *A*_i.s._ are the integrated areas of the converted portion (Figure 6b) and of the incident spectrum, respectively.

The second one is the effective efficiency, φ_eff_, *i.e.*, the efficiency in conversion of the incident light power density *P* (=19.7 mW·cm^−2^) into chemical energy [6], calculated by the following equation:

(3) $$\phi_{\text{eff}}=\frac{r_{H_{2}}\times \Delta G{}^{\circ}}{A\times P}\times 100$$

where Δ*G*° is the standard Gibbs free energy of the water spitting reaction (237 kJ·mol^−1^); and *A* (9.6 cm^2^) is the irradiated area of the photoelectrode.

The two efficiencies can slightly differ one from the other because φ_exp_ is calculated form transient experiment (the IPCE curves are measured while scanning λ), whereas φ_eff_ is calculated from the *r*_H_2__ values obtained under full lamp irradiation. The obtained efficiency values reported in Table 2, are relatively low, in line with the fact that the irradiation source employed in photocatalytic water splitting tests mainly emits in the 400–450 nm range, *i.e.*, above the photoactivity threshold of our samples. Notably, similar efficiency values were obtained for the same sample, although they were calculated from two different experiments and approaches. However, it is interesting to notice that φ_eff_ < φ_exp_ for samples showing *r*_H_2__/*r*_O_2__ > 2.3.

## 4. Conclusions

This work demonstrates that a more straightforward interpretation of photocatalytic water splitting results can be obtained by comparing them with those of IPCE measurements. In fact, the increase of hydrogen production rate obtained with an increase in the film thickness should not be attributed to an increased photocatalyst amount, but rather to a red-shift in photoactivity threshold in accordance with the increased amount of rutile phase.

## Figures and Tables

**Table 1:** Film thickness, crystal phase composition, and band gap of the investigated TiO_2_ coatings.

Deposition Time (h)	Film Thickness (nm) ^1^	Anatase (wt.%)	Rutile (wt.%)	Band Gap (eV)
1	320	54	46	3.30
3	1066	31	69	3.18
6	1823	30	70	3.05
8	3030	28	72	3.05

^1^ Calculated by the UV-vis-NIR DRS spectra.

**Table 2:** Effect of the deposition time on the photocatalytic performance of water splitting.

Deposition Time (h)	*r*_H_2__ (μmol·h^−1^)	*r*_O_2__ (μmol·h^−1^)	*r*_H_2__/*r*_O_2__	φ_exp_	φ_eff_
1	72.94 ± 0.06	31.62 ± 0.03	2.3	2.3	2.5
3	100.15 ± 0.09	40.3 ± 0.1	2.5	4.0	3.5
6	212.30 ± 0.03	98.4 ± 0.1	2.2	7.1	7.3
8	52.35 ± 0.08	21.81 ± 0.05	2.4	3.0	2.2

## Abbreviations

The following abbreviations are used in this manuscript:

RF	Radio frequency
XRD	X-ray diffraction
SEM	Scanning electron microscopy
DRS	Diffuse reflectance spectroscopy
IPCE	Incident photon to current efficiency

## References

[1] Li J., Wu N. (2015). Semiconductor-based photocatalysts and photoelectrochemical cells for solar fuel generation: A review. Catal. Sci. Technol..

[2] Lianos P. (2011). Production of electricity and hydrogen by photocatalytic degradation of organic wastes in a photoelectrochemical cell. The concept of the Photofuelcell: A review of a re-emerging research field. J. Hazard. Mater..

[3] Horiuchi Y., Toyao T., Takeuchi M., Matsuoka M., Anpo M. (2013). Recent advances in visible-light-responsive photocatalysts for hydrogen production and solar energy conversion—From semiconducting TiO_2_ to MOF/PCP photocatalysts. Phys. Chem. Chem. Phys..

[4] Selli E., Chiarello G.L., Quartarone E., Mustarelli P., Rossetti I., Forni L. (2007). A photocatalytic water splitting device for separate hydrogen and oxygen evolution. Chem. Commun..

[5] Chiarello G.L., Zuliani A., Ceresoli D., Martinazzo R., Selli E. (2016). Exploiting the photonic crystal properties of TiO_2_ nanotube arrays to enhance photocatalytic hydrogen production. ACS Catal..

[6] Chen Z., Jaramillo T.F., Deutsch T.G., Kleiman-Shwarsctein A., Forman A.J., Gaillard N., Garland R., Takanabe K., Heske C., Sunkara M. (2010). Accelerating materials development for photoelectrochemical hydrogen production: Standards for methods, definitions, and reporting protocols. J. Mater. Res..

[7] Molinari R., Marino T., Argurio P. (2014). Photocatalytic membrane reactors for hydrogen production from water. Int. J. Hydrog. Energy.

[8] Pessoa R.S., Fraga M.A., Santos L.V., Massi M., Maciel H.S. (2014). Nanostructured thin films based on TiO_2_ and/or SiC for use in photoelectrochemical cells: A review of the material characteristics, synthesis and recent applications. Mater. Sci. Semicond. Process..

[9] Liao C.-H., Huang C.-W., Wu J.C.S. (2012). Novel dual-layer photoelectrode prepared by RF magnetron sputtering for photocatalytic water splitting. Int. J. Hydrog. Energy.

[10] Tealdi C., Quartarone E., Galinetto P., Grandi M.S., Mustarelli P. (2013). Flexible deposition of TiO_2_ electrodes for photocatalytic applications: Modulation of the crystal phase as a function of the layer thickness. J. Solid State Chem..

[11] Binetti E., El Koura Z., Patel N., Dashora A., Miotello A. (2014). Rapid hydrogenation of amorphous TiO_2_ to produce efficient H-doped anatase for photocatalytic water splitting. Appl. Catal. A Gen..

[12] Ebrahimi A., Kitano M., Iyatani K., Horiuchi Y., Takeuchi M., Matsuoka M., Anpo M. (2012). Effect of the sputtering parameters on the physical properties and photocatalytic reactivity of TiO_2_ thin films prepared by an RF magnetron sputtering deposition method. Res. Chem. Intermed..

[13] Dholam R., Patel N., Adami M., Miotello A. (2009). Hydrogen production by photocatalytic water-splitting using Cr- or Fe-doped TiO_2_ composite thin films photocatalyst. Int. J. Hydrog. Energy.

[14] Wu M., Liu J., Jin J., Wang C., Huang S., Deng Z., Li Y., Su B.L. (2014). Probing significant light absorption enhancement of titania inverse opal films for highly exalted photocatalytic degradation of dye pollutants. Appl. Catal. B Environ..

[15] El Koura Z., Patel N., Edla R., Miotello A. (2014). Multilayer films of indium tin oxide/TiO_2_ codoped with vanadium and nitrogen for efficient photocatalytic water splitting. Int. J. Nanotechnol..

[16] Rahman M., Dang B.H.Q., McDonnell K., MacElroy J.M.D., Dowling D.P. (2012). Effect of doping (C or N) and co-doping (C + N) on the photoactive properties of magnetron sputtered titania coatings for the application of solar water-splitting. J. Nanosci. Nanotechnol..

[17] Wang C., Hu Q.Q., Huang J.Q., Deng Z.H., Shi H.L., Wu L., Liu Z.G., Cao Y.G. (2014). Effective water splitting using N-doped TiO_2_ films: Role of preferred orientation on hydrogen production. Int. J. Hydrog. Energy.

[18] Fakhouri H., Pulpytel J., Smith W., Zolfaghari A., Mortaheb H.R., Meshkini F., Jafari R., Sutter E., Arefi-Khonsari F. (2014). Control of the visible and UV light water splitting and photocatalysis of nitrogen doped TiO_2_ thin films deposited by reactive magnetron sputtering. Appl. Catal. B Environ..

[19] Kitano M., Iyatani K., Tsujimaru K., Matsuoka M., Takeuchi M., Ueshima M., Thomas J.M., Anpo M. (2008). The effect of chemical etching by HF solution on the photocatalytic activity of visible light-responsive TiO_2_ thin films for solar water splitting. Top. Catal..

[20] Matsuoka M., Kitano M., Fukumoto S., Iyatani K., Takeuchi M., Anpo M. (2008). The effect of the hydrothermal treatment with aqueous NaOH solution on the photocatalytic and photoelectrochemical properties of visible light-responsive TiO_2_ thin films. Catal. Today.

[21] Rietveld H.M. (1969). A profile refinement method for nuclear and magnetic structures. J. Appl. Crystallogr..

[22] Bish D.L., Howard S.A. (1988). Quantitative phase analysis using the Rietveld method. J. Appl. Crystallogr..

[23] Altomare A., Burla M.C., Giacovazzo C., Guagliardi A., Moliterni A.G.G., Polidori G., Rizzi R. (2001). Quanto: A Rietveld program for quantitative phase analysis of polycrystalline mixtures. J. Appl. Crystallogr..

[24] Lazzeri M., Vittadini A., Selloni A. (2001). Structure and energetics of stoichiometric TiO_2_ anatase surfaces. Phys. Rev. B.

[25] Barnard A.S., Curtiss L.A. (2005). Prediction of TiO_2_ nanoparticle phase and shape transitions controlled by surface chemistry. Nano Lett..

[26] Ramamoorthy M., Vanderbilt D., King-Smith R.D. (1994). First-principles calculations of the energetics of stoichiometric TiO_2_ surfaces. Phys. Rev. B.

[27] Kormann C., Bahnemann D.W., Hoffmann M.R. (1988). Preparation and characterization of quantum-size titanium dioxide. J. Phys. Chem..

[28] Chiarello G.L., Di Paola A., Palmisano L., Selli E. (2011). Effect of titanium dioxide crystalline structure on the photocatalytic production of hydrogen. Photochem. Photobiol. Sci..

[29] Ekimov A.I., Efros A.L., Onushchenko A.A. (1985). Quantum size effect in semiconductor microcrystals. Solid State Commun..

